# A Genome-Wide Association Study for Regulators of Micronucleus Formation in Mice

**DOI:** 10.1534/g3.116.030767

**Published:** 2016-05-27

**Authors:** Rebecca E. McIntyre, Jérôme Nicod, Carla Daniela Robles-Espinoza, John Maciejowski, Na Cai, Jennifer Hill, Ruth Verstraten, Vivek Iyer, Alistair G. Rust, Gabriel Balmus, Richard Mott, Jonathan Flint, David J. Adams

**Affiliations:** * Experimental Cancer Genetics, The Wellcome Trust Sanger Institute, Hinxton, Cambridgeshire CB10 1SA, UK; † Wellcome Trust Centre for Human Genetics, Oxford OX3 7BN, UK; ‡ Laboratory for Cell Biology and Genetics, The Rockefeller University, New York, New York 10065; § Microbial Pathogenesis, The Wellcome Trust Sanger Institute, Hinxton, Cambridgeshire CB10 1SA, UK; ** Tumour Profiling Unit, The Institute of Cancer Research, London SW3 6JB, UK; †† The Wellcome Trust/Cancer Research UK Gurdon Institute, University of Cambridge, CB2 1QN, UK; ‡‡ UCL Genetics Institute, University College London, WC1E 6BT, UK; §§ Laboratorio Internacional de Investigación sobre el Genoma Humano, Universidad Nacional Autónoma de México, Campus Juriquilla, Boulevard Juriquilla 3001, Santiago de Querétaro 76230, Mexico

**Keywords:** Micronuclei, genomic instability, genome-wide association study (GWAS), Outbred mice, genetic mapping

## Abstract

In mammals the regulation of genomic instability plays a key role in tumor suppression and also controls genome plasticity, which is important for recombination during the processes of immunity and meiosis. Most studies to identify regulators of genomic instability have been performed in cells in culture or in systems that report on gross rearrangements of the genome, yet subtle differences in the level of genomic instability can contribute to whole organism phenotypes such as tumor predisposition. Here we performed a genome-wide association study in a population of 1379 outbred Crl:CFW(SW)-US_P08 mice to dissect the genetic landscape of micronucleus formation, a biomarker of chromosomal breaks, whole chromosome loss, and extranuclear DNA. Variation in micronucleus levels is a complex trait with a genome-wide heritability of 53.1%. We identify seven loci influencing micronucleus formation (false discovery rate <5%), and define candidate genes at each locus. Intriguingly at several loci we find evidence for sexual dimorphism in micronucleus formation, with a locus on chromosome 11 being specific to males.

Genomic instability is a key hallmark of nearly all cancer cells (Hanahan and Weinberg 2011), and patients carrying loss-of-function mutations in components of the DNA damage response (DDR) machinery, including *BRCA1*, *BRCA2*, *ATM*, *BUB1*, and *RAD51*, are highly predisposed to tumorigenesis due to a significant increase in their basal somatic mutation rate (Watson *et al.* 2013). First-degree relatives of mutation carriers have also been shown to have a higher cancer incidence, suggesting that the link between mutations in DDR genes and cancer is heritable. In the general population, genomic instability has been linked to tumor incidence, with higher levels being associated with malignancies such as lung and prostate cancer, and tumors of the skin and brain (Bonassi *et al.* 2011; Forsberg *et al.* 2014). Furthermore, sequencing and genome-wide association studies have linked variants in DNA repair genes such as *CHEK2*, *ATM*, *RAD50*, *BRIP1*, and *PALB2* with breast cancer disease risk (Rahman 2014), highlighting the importance of DNA repair in tumor predisposition. In the same way, genes that regulate cell division and chromosome segregation also prevent genomic instability, and their mutation has been linked to tumorigenesis (Godinho and Pellman 2014; Lee 2014). Micronuclei have also been linked to chromothripsis (Zhang *et al.* 2015), the phenomenon by which up to thousands of clustered chromosomal rearrangements occur in a single event in localized and confined genomic regions.

One test to assess genomic instability *in vivo* is the micronucleus assay (Evans *et al.* 1959), which enumerates the number of peripheral erythrocytes that carry DNA. Since the nucleus is expelled during erythropoiesis, the presence of micronuclei in these cells is easily quantified and indicates that DNA damage occurred prior to enucleation. The frequency of micronucleated cells indicates the basal level of somatic genomic instability. However, it is unknown whether the presence and extent of micronuclei are heritable traits, and therefore under genetic control. Understanding their genetic architecture may therefore indicate pathways by which genetic instability and cancer arise.

To identify genetic mediators of micronucleus formation *in vivo* we used an outbred mouse population. Performing these studies in the mouse has several distinct advantages: First, in mice, micronucleated erythrocytes are not removed by the spleen meaning that micronucleus formation can be measured with high sensitivity and accuracy (Balmus *et al.* 2015). In humans these cells are rapidly cleared from the circulation. Second, functional studies can be followed up in the mouse by use of knockout or clustered regularly interspaced short palindromic repeats (CRISPR) technology (Wang *et al.* 2013), and thus candidate causal variants/genes can be assessed *in vivo*. Lastly, since multiple aspects of whole animal physiology, such as metabolism and endocrine function, may influence micronucleus formation we can also look for the contribution of these factors to micronucleus levels, which is not possible using *in vivo* culture systems.

Genetic mapping in the mouse using conventional crosses suffers from poor resolution, which presents a challenge when trying to identify the causal genes (Yalcin *et al.* 2010). To circumvent this issue we used a Swiss Webster outbred stock Crl:CFW(SW)-US_P08 (hereafter CFW) more suited to high-resolution mapping. This stock carries a limited number of segregating alleles at each locus, and a large number of recombination events leading to rapid linkage disequilibrium decay (Yalcin *et al.* 2010). We measured micronucleus levels and genotypes in 1379 CFW mice that were part of a larger multiphenotype study (Nicod *et al.* 2016), and for which genotypes were already available, to show that this is a heritable polygenic trait. We map numerous associated quantitative trait loci (QTL) and identify potential causal genes, as a prelude to further functional investigation.

## Materials and Methods

### Study animals and phenotyping

All mice [Crl:CFW(SW)-US_P08] were purchased from Charles River, Portage, at 4–7 wk of age and shipped to MRC Harwell, Oxfordshire, UK. Mice were housed in IVC cages (three per cage) on an *ad-libitum* diet and at 16 wk of age started a 4-wk phenotyping pipeline where behavioral and physiological measures were collected. This phenotyping pipeline and the data collected are described elsewhere (Nicod *et al.* 2016). At 20 wk of age mice were weighed and killed between 8 am and 12 pm, after overnight food restriction, and blood was collected by cardiac puncture into EDTA-coated vials. Full blood count analysis was performed with a Siemens Advia 2120 hematology analyzer using 200 µl of whole blood. In parallel a 50 µl aliquot of whole blood was collected into heparin solution, fixed in ice-cold methanol and stored at −80° until micronucleus levels were measured by flow cytometry as described previously (Balmus *et al.* 2015).

Analysis of micronucleus levels was performed using the R statistical analysis software package using purpose-written software available on request from the authors. Outliers, defined as data points that were more than three standard deviations from the mean, were excluded. The effects of covariates such as sex, body weight, and batch on micronucleus level were assessed by analysis of variance (ANOVA), with each explaining more than 1% of the variance at a significant level (*P* < 0.05) being included in a linear regression model from which residual measures were obtained. The linear model used for the micronucleus measure was the following: Micronucleus_level ∼1 + Sex + (1|Batch) + Year_of_measure. The residuals were quantile-normalized and used for genome-wide association testing. For the sex-specific genome-wide association testing, sex was omitted from the covariates in the modeling of the micronucleus measure.

### Studies in knockout lines

We analyzed knockouts for the genes *Trex1*, *Nfkbil1*, *Trub2*, and *H2-Eb1* (*Trex1^tm1(KOMP)Wtsi^*, *Nfkbil1^tm1a(KOMP)Wtsi^*, *Trub2^tm2a(EUCOMM)Wtsi^*, and *H2-Eb1^tm1a(KOMP)Wtsi^*) coming from the Sanger Mouse Genetics Project (White *et al.* 2013). Blood from these animals was collected at 16 wk and analyzed as previously described (Balmus *et al.* 2015).

### Sequencing, variant calling, and genotype imputation

Nicod *et al.* (2016) provides full details of the sequencing and genotyping protocol deployed in this study. In summary, DNA was extracted from tissues collected at the time of death. Sequencing libraries of 95 barcoded DNA samples were pooled and 100 bp paired-end sequencing reads were generated, using one lane of a HiSeq (Illumina), per pool yielding 30 Gb of sequence data. Reads were mapped to the mouse mm10 reference genome and variants called using all chromosomes pooled together. Imputation of genotype probabilities was performed using STITCH (Davies *et al.* 2016), which models the chromosomes in the CFW mice as mosaics of a limited number of founder haplotypes. Optimization showed that the most probable number of founder haplotypes was 4. The catalog of segregating variation in the CFW population was derived from a ∼370X pileup of all mice, combined with the positions of known SNPs in the Mouse Genomes Project (Keane *et al.* 2011). We identified 7,073,398 SNPs in this way, at which we imputed genotype dosages using STITCH (Davies *et al.* 2016). After stringent postimputation quality control we retained 5,766,828 high-quality imputed SNPs for subsequent analysis. We used two quality control measures on selecting well imputed *vs.* poorly imputed SNPs: the *P*-value for violation of Hardy-Weinberg equilibrium *P*-value and IMPUTE2-style INFO scores, as described previously (Howie *et al.* 2009). The mean SNP-wise correlation (*r*^2^) with sites that were also polymorphic on a genotyping microarray (Yang *et al.* 2009) using 44 samples was 0.974 before QC and 0.981 after QC.

### Genome-wide association

Details of QTL mapping are fully described in Nicod *et al.* (2016). In brief, we identified a subset of 359,559 SNPs tagging the entire genome and used the imputed allele dosages at these loci to compute the genome-wide additive genetic relationship matrix (GRM). We tested for the association between each tagging SNP (represented by its imputed dosage) and the quantile-normalized residuals of the micronucleus and hematological measures as fixed effects in a mixed-model, controlling for relatedness and population structure using a GRM as random effect. We used a leave-one chromosome-out strategy in which the GRM used to test association of SNPs on a given chromosome was computed from all other autosomes. Statistical significance at each locus was measured by ANOVA, comparing the fit of the allele dosage model to the null model. We estimated the false discovery rate (FDR) by permutation and called QTL when at least one SNP had an FDR <5%. After the discovery phase using the 360 K tagging SNPs, the genetic analysis was repeated in a 20 Mb window around the mapped QTL using the complete set of SNPs, to determine confidence intervals at each QTL using a logP-drop method. The same analysis was repeated testing males and females separately. We next tested for gene-by-sex interaction at all QTL detected with all mice (males and females). Significance of the interaction effect at the QTL was determined by ANOVA, comparing the fit of the interaction model between sex and the dosage at the most strongly associated SNP (pheno ∼ geno × sex) to the direct additive effect model (pheno ∼ geno + sex).

### Variant functional annotations

Putative SNPs were annotated using Annotate Variation (ANNOVAR) (Wang *et al.* 2010) with gene annotations/proteins from the University of California, Santa Cruz (UCSC) mouse genome annotation database (mouse assembly GRCm38/mm10). Unless otherwise stated, version 73 of the Ensembl mouse genome annotation database (assembly GRCm38/mm10) and software were used.

### All SNPs

For each SNP position, a GERP sequence conservation score (Pollard *et al.* 2010) was obtained from the Ensembl Compara database based on the alignments of 36 eutherian mammals [from the EPO whole-genome multiple alignment pipeline (ref http://sep2013.archive.ensembl.org/info/genome/compara/epo_anchors_info.html)]. A score for sequence constraint was also reported derived from stretches of the local, multiple alignment around each SNP (ref http://sep2013.archive.ensembl.org/info/genome/compara/analyses.html#conservation).

Both coding and noncoding sequence nucleotide variants (SNVs) were analyzed using the Ensembl Variant Effect Predictor (VEP) (Yourshaw *et al.* 2015). For coding SNPs, SIFT (Ng and Henikoff 2003) was used to predict whether an amino acid substitution may influence protein function, based on sequence homology and the physical properties of amino acids. Using transcription factor binding site data (ref http://sep2013.archive.ensembl.org/Mus_musculus/Experiment/Sources?db=funcgen;ex=all;fdb=funcgen;r=17:46617590-46621119#ExperimentalMetaData), noncoding SNPs were assessed for potential disruption of binding sites.

### Coding SNPs

A more detailed analysis to that performed for all the SNPs was performed for nonsynonymous SNPs predicted by ANNOVAR. GERP (Pollard *et al.* 2010) and sequence conservation scores were obtained as described previously (Yang and Wang 2015). In addition, text-based alignments were obtained from Ensembl EPO alignments of 13 eutherian mammals (Yates *et al.* 2016). A region of 10 nucleotides up- and down-stream for each SNP was specified, and the alignments across all potential 13 mammals were extracted for this 21-nucleotide region. Not all local regions had an alignment as some were unique to mouse.

With respect to protein-based analyses, VEP was configured, to report whether a coding SNP lay within a protein domain. As before, SIFT consequences (Ng and Henikoff 2003) were reported for each SNP, if available. In the case of multiple SNP predictions for a single SNP, the most deleterious prediction (lowest score) was reported.

In addition the effect of amino acid changes were assessed using the Grantham matrix (Grantham 1974). A SNP was classified as ‘conservative’ when it had a Grantham score of < 60. A score > 60 but < 100 was classified as ‘nonconservative.’ A SNP was classified as ’radical’ when it had a score >100. To obtain text-based protein alignments around a SNP, the following strategy was used: The ANNOVAR SNPs were predicted using UCSC protein data, identified using UCSC gene identifiers.

These UCSC gene identifiers were first used to link to Ensembl mouse transcript identifiers and then to Ensembl gene identifiers. The Ensembl gene identifiers were used to query the Ensembl Compara database for protein alignments across seven species; human, rat, dog, chicken, pig, cow, and platypus.

For computational efficiency the Ensembl Compara software pipeline selects a single (usually canonical) transcript for each gene on which to compute homologies across species. Similarity is determined by aligning protein alignments of genes. Therefore, for genes that have multiple translations, features of interest can occur in transcripts from which it is not possible to determine their context in terms of homology via Ensembl. This is the case here for coding SNPs reported by ANNOVAR. The set of UCSC proteins used included proteins that were not considered the canonical transcript by Ensembl and upon which homologies were computed. Hence for ANNOVAR, predicted coding SNPs reported on a transcript not precomputed in Ensembl, these were reported as ‘UCSC_protein_not_used_for_homology_in_Ensembl.’ Also there can be examples where the gene in which the SNP lies has no sequence homology to another species; these were tagged as ‘no_mouse_sequence_in_Ensembl_homology.’

For UCSC-based SNPs present in the Ensembl Compara database (Yates *et al.* 2016) the site of the amino acid was used to create an expanded peptide sequence of four amino acids up- and down-stream of the mutated site. This gave a nine amino acid sequence to match against alignments from other species. This sequence was shortened appropriately if the SNP site was close to the start or end of the protein in which it lay. The localized protein alignments were reported in text format for the seven species as listed previously.

### Analysis of micronuclei in TREX1 wild-type and mutant cells

RPE-1 derived cell lines were constructed as described previously (Maciejowski *et al.* 2015). Cells were plated onto 35 mm glass bottom dishes (MatTek) 48 hr before imaging. One hour before imaging cell culture media was replaced with phenol red-free DMEM/F12 medium. Live cell imaging was performed using a CellVoyager CV1000 spinning disk confocal system (Yokogawa, Olympus) equipped with 445, 488, and 561 nm lasers and a Hamamatsu 512 × 512 EMCCD camera. Pinhole size was 50 μm. Images were acquired at the indicated intervals using a UPlanSApo 60x/1.3 silicone oil objective with the correction collar set to 0.17. The pixel size in the image was 0.27 μm. The 617/73 emission filter was used for image acquisition of mCherry-tagged proteins. Sixteen-micrometer z-stacks were collected at 2.0 μm steps. Temperature was maintained at 37° in a temperature-controlled enclosure with CO_2_ support. Maximum intensity projection of z-stacks and adjustment of brightness and contrast were performed using Fiji software. Image stitching was done with the Fiji plugin Grid/Collection stitching (Preibisch *et al.* 2009) with 20% tile overlap, linear blending, a 0.30 regression threshold, a 2.50 max/avg. displacement threshold, and a 3.50 absolute displacement threshold. Images were cropped and assembled into figures using Photoshop CS5.1 (Adobe). Evaluation for statistical significance was carried out using ANOVA and the Kruskal-Wallis *post hoc* test.

### Ethics statement

The work described here was approved by the Oxford local ethics committee and was performed in accordance with Home Office Regulations, UK. A detailed description of the procedures is provided in Nicod *et al.* (2016).

### Data availability

The data and the results of the analysis described in this paper are available in an open-access database at http://outbredmice.org.

## Results

### Strain-specific and sex differences in micronucleus levels

**Figure 1:**
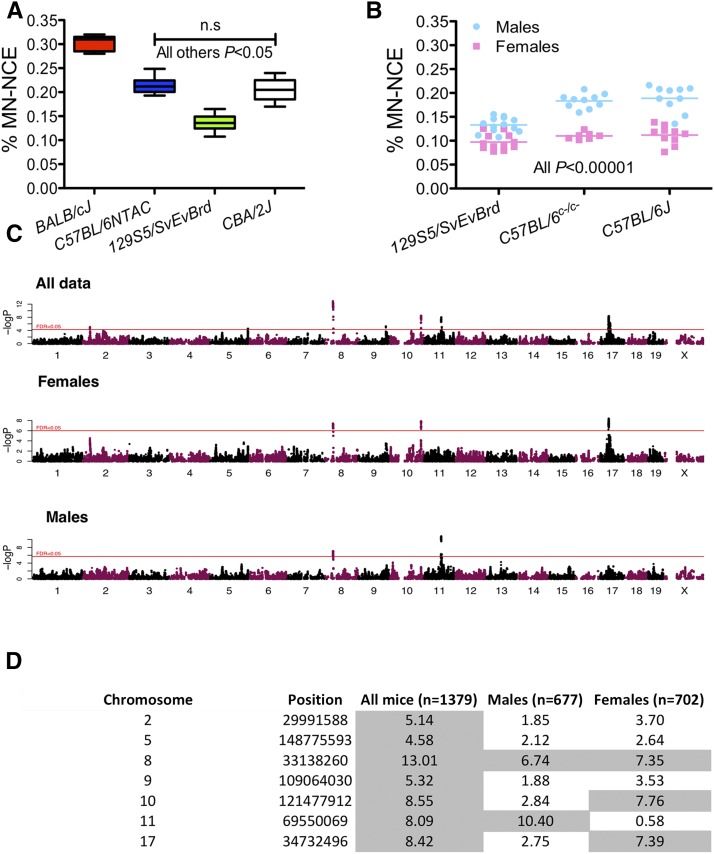
Micronucleus levels are genetically controlled. (A) Graph shows the percentage of micronucleated (MN) normochromatic erythrocytes (NCE) in males from four different inbred strains of mice. Boxplot shows the median and the min-max (whiskers) of at least six measurements for each strain. Data were analyzed by one-way ANOVA (*P* < 0.0001) followed by Tukey’s *post hoc* test (*P* < 0.05 for all combinations except C57BL/6NTAC and CBA/2J). (B) Micronucleus levels are influenced by sex in mice. Shown is the difference in micronucleus levels in males (blue) and females (pink) for three strains of inbred laboratory mice. Data were analyzed using the Student’s two-tailed *t*-test, *P* < 0.00001. (C) Manhattan plots of genome-wide analyses for micronucleus levels performed in all mice (top) or in female (middle) or male (bottom) mice only using the subset of tagging SNPs (*n* = 359,559). Horizontal red lines represent –log10 *P*-values at which QTL reach FDR < 5%. (D) QTL detected in all mice, males and females. Loci with FDR < 5% are shaded in gray. These QTL positions were defined using the above-mentioned 359,559 tagging SNPs. Shown are the maximum -log P values among tagging SNPs at each QTL.

To determine whether genetic diversity drives differences in micronucleus levels in mice, we first analyzed males from four inbred strains (Figure 1A) finding significant differences in micronucleus levels between strains (BALB/cJ > C57BL/6NTAC≅CBA/2J > 129S5SvEv^Brd^; *P* < 0.05; one-way ANOVA). Micronucleus levels were sexually dimorphic (Figure 1B), with females of two C57BL/6 substrains [C57BL/6J and C57BL/6^c-/c-^(C57BL/6-Tyr^C-Brd^)] and 129S5SvEv^Brd^ showing significantly lower micronucleus levels than male littermates (*P* < 0.00001; Student’s two-tailed *t*-test).

### Genetic mapping in outbred mice

We provide a detailed description and characterization of the CFW mouse population used in this study in Nicod *et al.* (2016). To map loci linked to micronucleus formation in the CFW we used a highly sensitive and reproducible high-throughput flow-cytometric micronucleus assay to score the frequency of micronucleated erythrocytes in blood obtained from 1485 unrelated CFW animals (733 males and 752 females) culled at 20 wk of age (Balmus *et al.* 2015). Initial analysis revealed micronucleus levels to be approximately normally distributed (Supplemental Material, Figure S1). Body weight and a full hematological profile were also measured at the time of blood collection. DNA was obtained from 1379 of these mice (677 males and 702 females) and, following sparse whole-genome sequencing at an average coverage of 0.15X (range 0.06X to 0.51X), genotype probabilities were imputed at 7,073,398 SNPs segregating within this population (Nicod *et al.* 2016). For the genetic analysis we retained 5,766,828 SNPs that passed a stringent postimputation quality control threshold [Impute2-like INFO score >0.4 and (in autosomes only) *P*-value for Hardy-Weinberg equilibrium *R*^2^ > 1 × 10^−6^].

To map QTL we used a subset of 359,559 SNPs tagging all other SNPs with a minor allele frequency (MAF) >0.1% at LD *R*^2^ > 0.98, thereby capturing the common genetic variability present in this population. The heritability of micronucleus levels in the CFW population, as estimated from the additive GRM based on these tagging SNPs, was 53.1% (SE 7.7%) (Nicod *et al.* 2016). Genetic mapping was performed using a mixed model by testing the association of each tagging SNP as a fixed effect with the level of micronuclei, using the same GRM as a random effect, and controlling for relevant covariates (see *Materials and Methods* and Nicod *et al.* 2016). This genome-wide analysis revealed seven QTL at an FDR < 5% (Figure 1, C and D). We then repeated the genetic analysis around each QTL using all nearby SNPs from the total catalog of 5,766,828, to fine-map and determine the confidence interval (CI) at each locus (see *Materials and Method*s).

**Figure 2:**
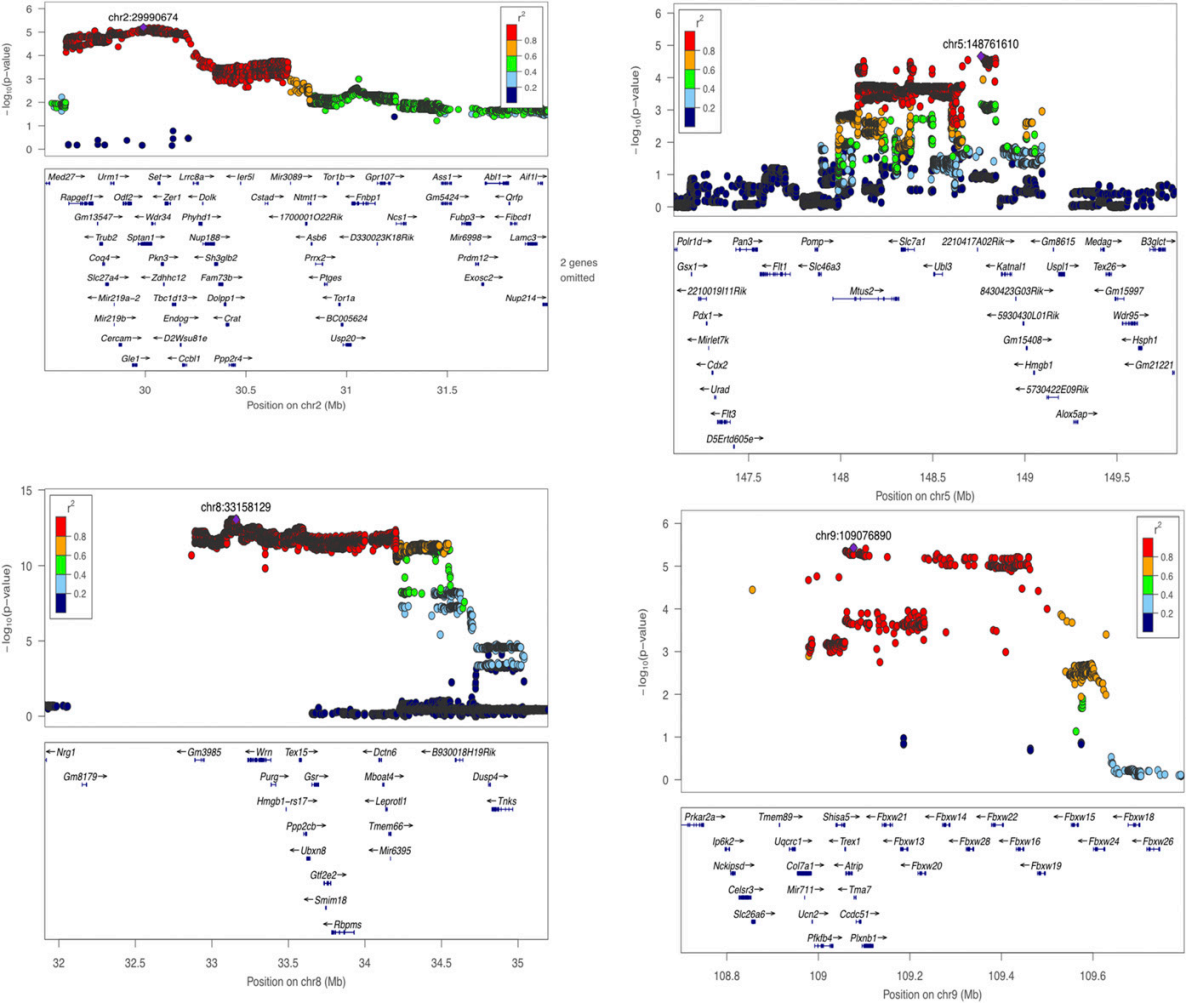
Genome-wide significant loci for micronucleus formation on chromosomes 2, 5, 8, and 9. The −log10 *P*-values of imputed single-nucleotide polymorphisms (SNPs) associated with micronucleus levels are shown on the Y axis. The X axis gives chromosome and position in megabases (Mb). Genes within the regions are shown in the bottom panels (for clarity, as indicated on the figure, some gene names have been omitted). Linkage disequilibrium of each SNP with top SNP, shown in large purple diamond, is indicated by its color. The plots were drawn using LocusZoom (Pruim *et al.* 2010).

**Figure 3:**
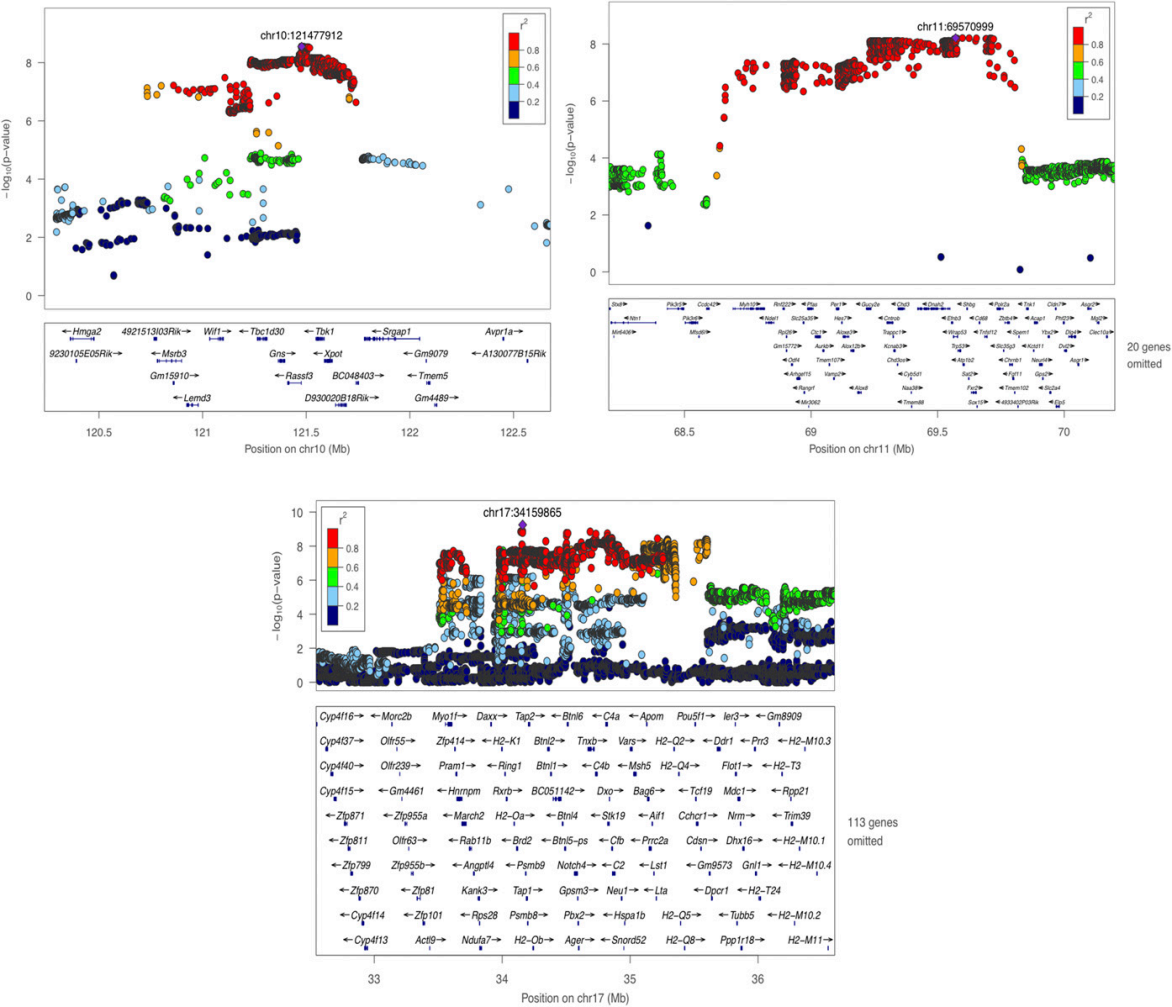
Genome-wide significant loci for micronucleus formation on chromosomes 10, 11 and 17. The −log10 *P*-values of imputed SNPs associated with micronucleus levels are shown on the left Y axis. The horizontal axis gives chromosome and position in megabases (Mb). Genes within the regions are shown in the bottom panels (for clarity, as indicated on the figure, some gene names have been omitted). Linkage disequilibrium of each SNP with top SNP, shown in large purple diamond, is indicated by its color. The plots were drawn using LocusZoom (Pruim *et al.* 2010).

Table 1 shows a summary of the seven loci identified and their 95% CI, with sizes ranging from 476 kb to 1.46 Mb (mean 905 kb). In total, the 95% CI include 197 coding genes, or an average of 28 genes identified at each QTL. The most significant locus was on chromosome 8 with a –log10 *P*-value of 13.05 (Table 1). At this locus the most significant SNP fell next to the Werner syndrome *Wrn* gene. *WRN* is a RecQ helicase having intrinsic 3′ to 5′ DNA helicase activity (Gray *et al.* 1997; Shen and Loeb 2000). It interacts with Ku70/80, and participates in DNA end processing. Defects in *WRN* are associated with premature aging, chromosomal instability, and tumorigenesis (Shen and Loeb 2000). Candidates at other loci include *Trp53*, *Rassf3*, and *Trub2*, all of which have established roles in the regulation of DNA repair/genomic stability (Smith and Fornace 1995; Zucchini *et al.* 2003; van der Weyden and Adams 2007) (Figure 2 and Figure 3).

**Table 1:** Genome-wide significant loci

Chromosome	Top SNP Position	Top SNP logP	FDR	Size 95% CI (bp)	Start Position 95% CI	End Position 95% CI	Number of Genes in 95% CI	Gene Names	MAF	β	Variance Explained (%)
2	29990674	5.21	0.017	661130	29606566	30267695	20	*Rapgef1*, *Gm13547*, *Trub2*, *Coq4*, *Slc27a4*, *Urm1*, *Cercam*, *Odf2*, *Gle1*, *Sptan1*, *Wdr34*, *Set*, *Pkn3*, *Zdhhc12*, *Zer1*, *Tbc1d13*, *Endog*, *D2Wsu81e*, *Ccbl1*, *Lrrc8a*	0.49	0.33	1.29
5	148761610	4.66	0.045	747871	148093705	148841575	3	*Mtus2*, *Slc7a1*, *Ubl3*	0.46	0.27	1.13
8	33158129	13.05	0	1312071	32889539	34201609	13	*Wrn*, *Purg*, *Tex15*, *Ppp2cb*, *Ubxn8*, *Gsr*, *Gtf2e2*, *Smim18*, *Rbpms*, *Dctn6*, *Mboat4*, *Leprotl1*, *Saraf*	0.38	0.59	4.46
9	109076890	5.43	0.015	624542	108855860	109480401	20	*Slc26a6*, *Tmem89*, *Uqcrc1*, *Col7a1*, *Ucn2*, *Pfkfb4*, *Shisa5*, *Trex1*, *Atrip*, *Tma7*, *Ccdc51*, *Plxnb1*, *Fbxw21*, *Fbxw13*, *Fbxw20*, *Fbxw14*, *Fbxw14*, *Fbxw22*, *Fbxw16*, *Fbxw19*	0.20	0.46	1.77
10	121477912	8.55	0	476002	121229924	121705925	6	*Tbc1d30*, *Gns*, *Rassf3*, *Tbk1*, *Xpot*, *D930020B18Rik*	0.50	0.46	2.98
11	69570999	8.21	0	1055268	68747919	69803186	49	*Myh10*, *Ndel1*, *Rnf222*, *Rpl26*, *Odf4*, *Arhgef15*, *Slc25a35*, *Rangrf*, *Pfas*, *Ctc1*, *Aurkb*, *2310047M10Rik*, *Tmem107*, *Vamp2*, *Per1*, *Hes7*, *Aloxe3*, *Alox12b*, *Alox8*, *Gucy2e*, *Cntrob*, *Trappc1*, *Kcnab3*, *Chd3*, *Cyb5d1*, *Naa38*, *Tmem88*, *Kdm6b*, *Dnah2*, *Efnb3*, *Wrap53*, *Trp53*, *Atp1b2*, *Shbg*, *Sat2*, *Fxr2*, *Sox15*, *Mpdu1*, *Cd68*, *Eif4a1*, *Senp3*, *Tnfsfm1 3*, *Tnfsf13*, *Tnfsf12*, *Polr2a*, *Slc35g3*, *Zbtb4*, *Chrnb1*, *Fgf11*	0.45	0.45	2.55
17	34159865	9.25	0	1456751	34145616	35602366	86	*H2-DMb2*, *H2-DMb1*, *Psmb9*, *Tap1*, *Psmb8*, *Tap2*, *H2-Ob*, *H2-Ab1*, *H2-Aa*, *H2-Eb1*, *H2-Eb2*, *Btnl2*, *Btnl1*, *BC051142*, *Btnl4*, *Btnl6*, *Notch4*, *Gpsm3*, *Pbx2*, *Ager*, *Rnf5*, *Agpat1*, *Egfl8*, *Ppt2*, *Prrt1*, *Fkbpl*, *Atf6b*, *Tnxb*, *C4b*, *Cyp21a1*, *Stk19*, *Dxo*, *Skiv2l*, *Nelfe*, *Cfb*, *C2*, *Zbtb12*, *Ehmt2*, *Slc44a4*, *Neu1*, *Hspa1b*, *Hspa1a*, *Hspa1l*, *Lsm2*, *Vars*, *Vwa7*, *Sapcd1*, *Msh5*, *Clic1*, *Ddah2*, *G6b*, *Ly6g6c*, *Ly6g6d*, *Ly6g6e*, *Ly6g6f*, *Abhd16a*, *Ly6g5c*, *Ly6g5b*, *Csnk2b*, *Gpank1*, *D17H6S53E*, *Apom*, *Bag6*, *Prrc2a*, *Aif1*, *Lst1*, *Ltb*, *Tnf*, *Lta*, *Nfkbil1*, *Atp6v1g2*, *Ddx39b*, *H2-D1*, *H2-Q1*, *H2-Q2*, *H2-Q4*, *H2-Q6*, *H2-Q7*, *H2-Q10*, *Pou5f1*, *Tcf19*, *Cchcr1*, *Psors1c2*, *Cdsn*, *2300002M23Rik*, *Sfta2*	0.38	0.74	3.16

Shown are the seven genome-wide significant loci for micronucleus levels and the genes within the 95% confidence intervals. The start and end positions of each QTL are provided, and the minor allele frequency (MAF), β, and effect size (variance explained) of the top scoring SNP. These QTL positions were defined using the entire collection of SNPs for higher mapping resolution. Note that the -logP value given in the table is the maximum among all imputed SNPs under the QTL, which is generally higher than that shown on Fig 1D.

Interestingly, after correcting for the effect of potential confounding variables (see *Materials and Methods*), we found small but highly significant positive correlations between micronucleus level and several hematological measures; the red blood cells distribution width (RDW, *P* = 2.55 *×* 10^−26^, Spearman *R*^2^ = 0.1), the hemoglobin concentration distribution width (HDW, *P* = 1.63 *×* 10^−12^, *R*^2^ = 0.04, Spearman), the mean cellular hemoglobin concentration (CHCM, *P* = 3.14*×*10^−11^, R^2^ = 0.04, Spearman), and the measure of blood hemoglobin (measHGB, *P* = 1.88 *×* 10^−6^, *R*^2^ = 0.02, Spearman). Elevation of RDW, which is a measure of the variance of red blood cell width, often occurs together with elevated HDW, which measures the variation in hemoglobin content of red blood cells. For example, iron deficiency may cause a reduction in hemoglobin production (elevated HDW), which causes smaller red blood cells (elevated RDW). Elevation of RDW and/or HDW is a characteristic of anemia, a condition that may be caused by genomic instability (O’Driscoll 2012), and mouse models of genome instability disorders sometimes display hematological abnormalities (Crossan *et al.* 2011; Nijnik *et al.* 2012). With this in mind we performed a genetic analysis with these hematological measures and discovered that measHGB and CHCM are both associated with the same locus on chromosome 5 [*P* = 3.67 × 10^−7^ and 5.05 × 10^−7^, respectively (Figure S2)]. We also mapped a micronucleus QTL at this locus, that contains only three genes within its 95% CI, including *Slc7a1*. Mice carrying a homozygous deleterious mutation of this gene die of anemia at birth with 50% fewer red blood cells and reduced hemoglobin levels (Perkins *et al.* 1997). Hence, *Slc7a1* is a potential causative gene for the hematological traits at this locus with a possible indirect effect on micronucleus levels. However, another gene within the QTL is the Microtubule Associated Tumor Suppressor Candidate 2 (*Mtus2*) (Jiang *et al.* 2009), which has been implicated in the function of the microtubule cytoskeleton (Ward *et al.* 2013).

### Erythropoietic micronucleus formation and sex

Consistent with our observation of differences between sexes in micronucleus levels, genetic mapping performed on each sex independently revealed that, when applying the same FDR < 5% threshold for QTL discovery, two loci (on Chrs 10 and 17) were detected only in females and one, on Chr 11, only in males (Figure 1C). We tested for gene-by-sex interactions at all QTL using the entire dataset and found that the locus on chromosome 11 shows a sex-specific effect on micronucleus formation in male mice (ANOVA *P =* 3.13 × 10^−4^). The QTL on Chr 8 is present in both sexes while the QTL on chromosomes 2, 5, and 9 do not reach genome-wide significance (FDR < 5%) when males and females are tested separately, presumably due to a lack of power (Figure 1D).

### Identification of candidate genes at QTL

Gene ontology (GO) analysis revealed four genes under the micronucleus QTLs (Table S1) with annotations associated with DNA repair: *Wrn*, *Hspa1b*, *Hspa1a*, and *Hspa1l* (GO 0006281). Other genes such as *Aurora kinase B* (*Aurkb*) have established roles in the regulation of processes such as the cell cycle (Adams *et al.* 2001), and links to processes such as mitosis, which might be expected to also result in elevated levels of micronuclei if dysregulated.

Our genotyping by low-coverage sequencing methodology means we have identified most high-frequency SNPs segregating in the CFW population, and can test them for association with the trait, opening up the possibility of identifying functional variant(s) at each QTL. From our set of 5,766,828 SNPs we annotated all variants in the 95% CI at each QTL provided they had a *P*-value of association with micronucleus levels <10^−3^ to ascertain if they could disrupt gene function, and thus potentially contribute to elevated micronucleus levels. We used a combination of approaches to assess the pathogenicity of variants by using ANNOVAR, applying GERP scores, and by performing a Grantham and SIFT analysis. We also used VEP from Ensembl (McCarthy *et al.* 2014). Table 2 shows a list of the top five scoring SNPs at each locus ranked by *P*-value; at some loci there were fewer than five or no coding SNPs that could be scored in this way.

**Table 2:** Pathogenicity analysis of variants at loci associated with elevated micronucleus levels

Mouse genomic location	Nearest gene	Consequence	Log *P* value	GERP Score	Grantham classification	Grantham score	SIFT class	Lowest SIFT score	EnsEMBL VEP class	EnsEMBL aa change	AA Change	Nuc Change
chr8:33576360-33576360	*Tex15*	A > T	12.48026273	−1.05	Radical	110	Tolerated	0.4	Downstream gene variant; missense variant	R/S	*R1939S*	*A5817T*
chr8:33268792-33268792	*Wrn*	G > A	12.45662832	−3.07	Radical	145	Tolerated	0.3	Missense variant	S/L	*S1021L*	*C3062T*
chr8:33557389-33557389	*Tex15*	A > C	12.43606688	3.19	Radical	110	Tolerated	0.44	NMD transcript variant; downstream gene variant; missense variant	S/R	*S160R*	*A478C*
chr8:33557366-33557366	*Tex15*	G > T	12.4319775	3.14	Radical	205	Tolerated	0.13	NMD transcript variant; downstream gene variant; missense variant	C/F	*C152F*	*G455T*
chr8:33546343-33546343	*Tex15*	C > G	12.42405548	3.56	Nonconservative	60	Deleterious; tolerated	0.02	NMD transcript variant; missense variant	A/G	*A94G*	*C281G*
chr17:34804124-34804124	*Cyp21a1*	A > G	8.239597012	0	Nonconservative	64	Deleterious	0.01	Downstream gene variant; missense variant; upstream gene variant	V/A	*V71A*	*T212C*
chr17:34711526-34711526	*Tnxb*	G > A	8.198665514	4.53	Radical	125	Deleterious; tolerated	0.05	Missense variant	G/R	*G397R*	*G1189A*
chr17:34711722-34711722	*Tnxb*	G > A	8.188940007	−9.05	Conservative	29	Deleterious	0.02	Missense variant	R/H	*R462H*	*G1385A*
chr17:34713138-34713138	*Tnxb*	T > A	8.185003889	−2.37	Radical	113	Tolerated	0.21	Missense variant	L/Q	*L550Q*	*T1649A*
chr17:35161042-35161042	*Prrc2a*	T > G	8.088956801	4.11	Conservative	15	Deleterious	0.02	Downstream gene variant; missense variant; upstream gene variant	M/L	*M249L*	*A745C*
chr11:69236603-69236603	*Gucy2e*	C > G	7.758017279	−9.38	Radical	125	Tolerated	0.46	Missense variant; upstream gene variant	G/R	*G15R*	*G43C*
chr10:121667433-121667433	*D930020B18Rik*	T > C	7.543972828	0.726	Radical	155	Tolerated	0.85	Missense variant	F/S	*F75S*	*T224C*
chr11:69047870-69047870	*Aurkb*	T > C	7.224042226	2.01	Radical	155	Tolerated	0.74	Missense variant; upstream gene variant	F/S	*F45S*	*T134C*
chr11:69134001-69134001	*Aloxe3*	G > A	6.687629704	3.92	Conservative	56	Deleterious	0.01	NMD transcript variant; missense variant; upstream gene variant	G/S	*G341S*	*G1021A*
chr11:69107564-69107564	*Per1*	C > T	6.656511366	2.92	Nonconservative	64	Deleterious	0.01	Downstream gene variant; missense variant	A/V	*A1014V*	*C3041T*
chr9:109061596-109061596	*Atrip*	A > T	5.330403284	−0.969	Radical	113			NMD transcript variant; downstream gene variant; missense variant; synonymous variant; upstream gene variant	L/Q	*NA*	*NA*
chr9:109073661-109073661	*Atrip*	T > A	5.271500634	0.625	Radical	152	Deleterious	0.03	NMD transcript variant; downstream gene variant; missense variant; splice region variant; upstream gene variant	D/V	*NA*	*NA*
chr9:109432436-109432436	*Fbxw16*	A > G	5.023943697	0	Radical	155	Tolerated	0.56	Missense variant	F/S	*F454S*	*T1361C*
chr2:30174134-30174134	*D2Wsu81e*	C > T	4.890540776	4.1	Radical	125	Tolerated	0.34	Downstream gene variant; missense variant	G/R	*G373R*	*G1117A*
chr2:30174134-30174134	*D2Wsu81e*	C > T	4.890540776	4.1	Radical	125	Tolerated	0.34	Downstream gene variant; missense variant	G/R	*G373R*	*G1117A*
chr2:29702527-29702527	*Rapgef1*	G > T	4.806085521	1.45	Conservative	24	Deleterious; tolerated	0.02	NMD transcript variant; missense variant	Q/H	*Q510H*	*G1530T*
chr2:29702527-29702527	*Rapgef1*	G > T	4.806085521	1.45	Conservative	24	Deleterious; tolerated	0.02	NMD transcript variant; missense variant	Q/H	*Q510H*	*G1530T*
chr2:29788325-29788325	*Coq4*	G > T	4.571110541	0	Radical	102	Tolerated	0.32	NMD transcript variant; missense variant; upstream gene variant	R/L	*R14L*	*G41T*
chr9:109060491-109060491	*Atrip*	T > G	3.712727076	0.625	Conservative	15	Deleterious; tolerated	0.03	NMD transcript variant; downstream gene variant; missense variant; upstream gene variant	M/L	*M688L*	*A2062C*
chr5:148288927-148288927	*Mtus2*	C > A	3.699186538	0	Radical	110			NMD transcript variant; missense variant; synonymous variant	S/R	*NA*	*NA*
chr5:148288935-148288935	*Mtus2*	T > C	3.692862836	0	Radical	145			NMD transcript variant; missense variant; synonymous variant	L/S	*NA*	*NA*
chr9:109058117-109058117	*Trex1*	G > C	3.215586866	−1.25	Radical	125	Tolerated	0.18	Downstream gene variant; missense variant	R/G	*R269G*	*C805G*

Shown are the top five scoring variants at each of the seven genome-wide significant loci ranked by –log10 *P*-value. See *Materials and Methods* for a description of the approach used for variant annotation.

**Figure 4:**
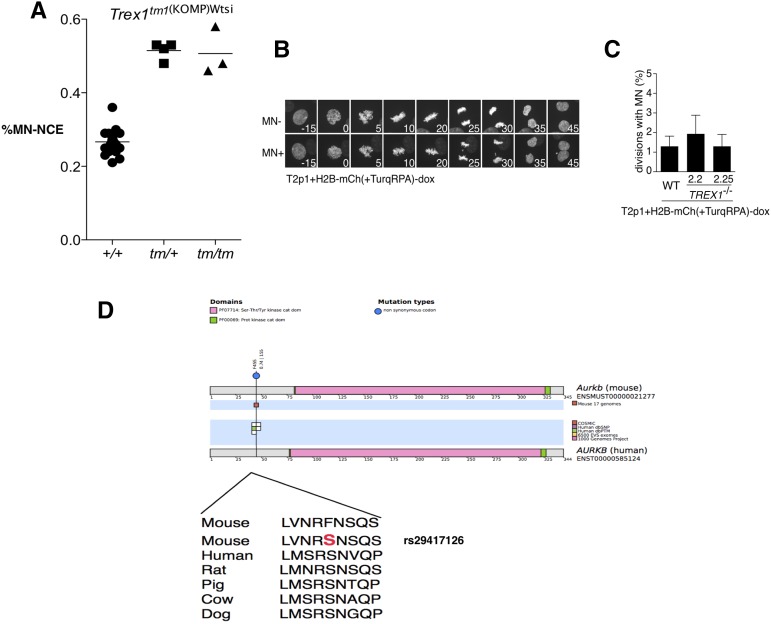
Candidate genes from genome-wide significant loci. (A) Frequency of propidium iodide positive, micronucleated (MN) normochromatic erythrocytes (NCE) in wild-type (+/+), heterozygote (tm1/+), and homozygote (tm/tm) *Trex1* knockout male mice. Each circle, square, or triangle indicates an individual mouse. Mutant mice had significantly elevated MN-NCE when compared to wild-type control mice (Student’s two-tailed *t*-test; *P* < 0.0001), but heterozygous and homozygous mice showed comparable levels of MN-NCE. (B and C) Micronucleus formation in human *TREX1* null and wild-type control cells. Chromatin was labeled with H2B-mCherry. The data shown are the result of three independent experiments where >100 mitoses were counted. Cell lines 2.2 and 2.5 are *TREX1* null RPE-1 cells. For a full description of the lines used in these experiments see Maciejowski *et al.* (2015). Wild type (WT) refers to an isogenic control. (D) Schematic to show alignment of mouse and human AURKB proteins. A candidate single nucleotide polymorphism (SNP) in *Aurkb* (rs29417126) falls into a highly conserved residue of AURKB that is known to be mitotically phosphorylated.

We found missense variants with strong predicted deleterious effects in the *Wrn*, *Atrip*, *Trex1*, and *Aurkb* genes, which, as noted above, have annotated roles in the regulation of genomic stability making these variants high-value candidates. For *Aurkb* the SNP identified (rs29417126) results in a F45S change, and falls into a highly conserved region of the protein (Figure 4). We projected this position onto the human AURKB protein revealing that it falls into a residue previously found to be post-translationally modified/phosphorylated (Daub *et al.* 2008). Indeed, this position has been found to be mitotically phosphorylated, and thus is likely to play a regulatory role in AURKB function. Thus *Aurkb* is a likely gene responsible for the association at the QTL on chromosome 11. Importantly this QTL also includes the gene *Trp53*, which is involved in the cellular response to DNA repair, and thus is another potential candidate at this locus. However, we did not identify any missense coding variant on the *Trp53* gene in the CFW population, an observation in line with the absence of variants in the classical inbred strains (Keane *et al.* 2011). However, it is possible that a causal regulatory variant that controls expression of *Trp53* may yet be identified.

We next took advantage of both published and novel mouse knockout data to study genes within our QTL intervals. Over 1800 mammalian phenotype (MP) terms have been assigned to the 197 genes within the QTL, including some associated with genomic instability, spindle abnormalities, and defects in replication (Table S1). For many of the genes, phenotypes/functions associated with genomic instability have been assigned (Table S2). We then attempted to find genes associated with elevated levels of micronuclei. To do this we searched for live mice generated by the Wellcome Trust Sanger Mouse Genetics Project (White *et al.* 2013). This analysis revealed four strains (*Trex1*, *Nfkbil1*, *Trub2*, and *H2-Eb1*) available for testing using the micronucleus assay. Our analysis of potentially pathogenic variants within QTL (Table 2) revealed a nonsynonymous change within *Trex1* (R269G; rs386972414) as a candidate variant. TREX1 is a major 3′-5′ DNA exonuclease linked to systemic lupus erythematosus and also Aicardi–Goutières syndrome (Fauré *et al.* 1999; Crow *et al.* 2000). Analysis of blood from *Trex1^+/−^* and *Trex1^−/−^* mice revealed significantly elevated levels of DNA-positive red blood cells, making this gene a strong candidate at this locus (Figure 2 and Figure 4). The micronucleated erythrocyte frequency for *Nfkbil1*, *Trub2*, and *H2-Eb1* mutants was not significantly different to that of wild-type control mice (Figure S3).

The identification of elevated levels of micronuclei in *Trex1* mice was unexpected given the role of this gene in single-stranded DNA processing but not directly in DNA repair or chromosome segregation. We next used time lapse microscopy of micronuclei in isogenic human cell lines (RPE-1) in which chromatin (H2B) was labeled with mCherry in the context of *TREX1* disruption, and in a matched control (*TREX1* wild-type) line (Figure 4). Analysis of 808 cell divisions from *TREX1* mutant cells and 405 from wild-type cells revealed no difference in the frequency of large micronuclei, a result in keeping with the observation that *Trex1* mutant mice do not show elevated levels of spontaneous mutation in the Big Blue assay (Morita *et al.* 2004). We conjecture that the elevated frequency of RBCs staining with propidium iodide may be the result of the accumulation of single-stranded DNA fragments previously reported to accumulate in tissues from *Trex1* mutants (Morita *et al.* 2004), rather than bulky micronuclei resulting from chromosomal breaks or whole chromosome loss. This suggests that the micronucleus assay used here is capable of identifying genes with a range of DNA process functions beyond those involved in DNA repair/chromosome segregation. It is important to note that *Trex1* abuts *Atrip*, a known DNA repair gene, and we cannot exclude an indirect effect of the targeting event on *Atrip* gene function.

## Discussion

We report an *in vivo* genetic screen for mediators of micronucleus formation and identify seven loci that reach genome-wide significance. At these loci we mapped genes that have established roles in DNA repair or the regulation of the cell cycle, and we annotated variants at these loci to identify possible candidates associated with elevated micronucleus levels. One locus also affects two hematological measures and contains a gene (*Slc7a1*) causing severe anemia in the mouse. Further, functional evidence *in vivo* in a mouse mutant supports the role of *Trex1* in the formation of extranuclear DNA.

We found evidence for a role of sex in the formation of micronuclei, with a locus on chromosome 11 containing the *Aurkb* gene being male-specific. In the case of erythrocyte micronucleus levels it seems unlikely that this phenotype is mediated by sex hormones or anatomical differences between male and female mice, although these factors could possibly contribute. Interestingly, men have a higher incidence of and mortality rate from sex-unspecific cancers, a fact that is unexplained by known risk factors (Cook *et al.* 2011; Edgren *et al.* 2012). Recently, mosaic loss of the chromosome Y in peripheral blood cells, which indicates loss of Y in hematopoietic progenitor cells, was associated with reduced survival and a higher risk of cancer in men (Forsberg *et al.* 2014). In CFW mice we found elevated micronucleus levels in males and find a sex-specific locus controlling this trait, further highlighting the role of sex in predisposition to tumorigenesis.

What might explain the differences in micronucleus levels in male and female mice that we observed? It has previously been shown that the regulatory genome is sexually dimorphic (Yang *et al.* 2006) with as many as 70% of transcripts showing sex-specific differences in expression (Yang *et al.* 2006), and recent work has shown that the expression of several DNA repair proteins is influenced by sex. Sexually dimorphic regulation of DNA repair genes could have evolved to support processes such as meiosis, which is regulated differently between the sexes (Morelli and Cohen 2005). In addition to the locus on chromosome 11 six other loci containing both known and novel candidate DNA repair genes were identified. It is important to note that while micronuclei are a marker of genomic instability they also measure the genotoxic effects of disease processes. For example, micronuclei are elevated in autoimmune disorders such as systemic lupus erythematosus and thus may be associated with other traits/phenotypes defined in this outbred population (Al-Rawi *et al.* 2014). Future studies will involve validating candidate variants at these loci, for example using CRISPR-mediated gene editing.

Collectively, this work reveals the landscape of micronucleus formation in the mouse and the value of studying traits in an outbred mouse population, and at the whole organism level.

## Supplementary Material

Supplemental Material
