# Supplementary material for: A Genome-Wide Association Study for Regulators of Micronucleus Formation in Mice
Source: G3 (Bethesda). 2016 May 27;6(8):2343–54. doi: 10.1534/g3.116.030767 (PMC4978889; doi:10.1534/g3.116.030767)
Supplement: Supplemental Material [file supp_6_8_2343__index.html]

A Genome-Wide Association Study for Regulators of Micronucleus Formation in Mice — Supplemental Material 

# A Genome-Wide Association Study for Regulators of Micronucleus Formation in Mice

## Supplemental Material for McIntyre, *et al*, 2016

**Files in this Data Supplement:**

- Figure S3 - Micronucleus analysis of knockout mouse lines. The genes shown are candidate genes from within QTL intervals identified in this screen. %MN-NCE refers to percentage of micronucleated normochromatic erythrocytes. (.pdf, 19 KB)
- Figure S1 - Distribution of micronucleus frequencies (%MN-NCE) for all outbred mice analysed in this study. %MN-NCE refers to percentage of micronucleated normochromatic erythrocytes. (.tiff, 54 KB)
- Figure S2 - QTL analysis of the hematological measures measHGB and CHCM at the same locus on chromosome 5 (P = 3.67x10^-7^ and 5.05x10^-7^, respectively). (.tiff, 1,299 KB)
- Table S1 - Gene ontology (GO) analysis of genes in QTL intervals identified in this study. The analysis shown was performed using MGI batch query. (.xlsx, 113 KB)
- Table S2 - MP term analysis of genes in QTL intervals identified in this study. The analysis shown was performed using MGI batch query. (.xlsx, 240 KB)
