# Supplementary figures and images for: A Genome-Wide Association Study for Regulators of Micronucleus Formation in Mice
Source: G3 (Bethesda). 2016 May 27;6(8):2343–54. doi: 10.1534/g3.116.030767 (PMC4978889; doi:10.1534/g3.116.030767)

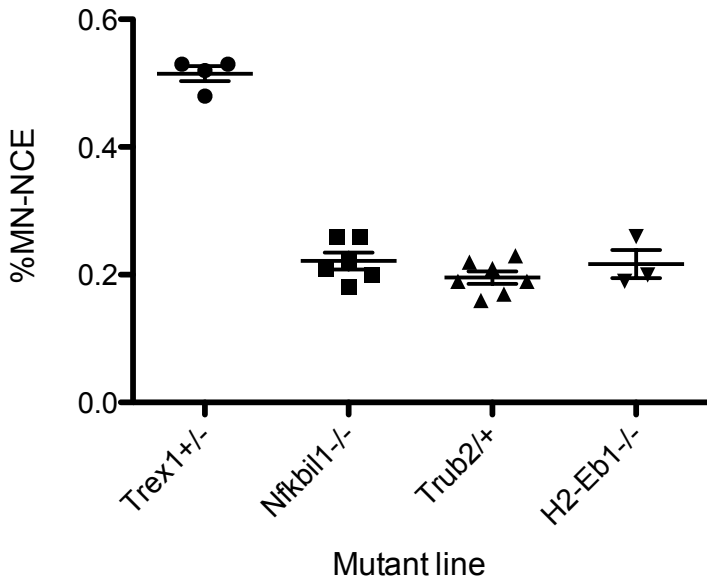

Supplement: Supplemental Material [file supp_g3.116.030767_FigureS3.pdf]

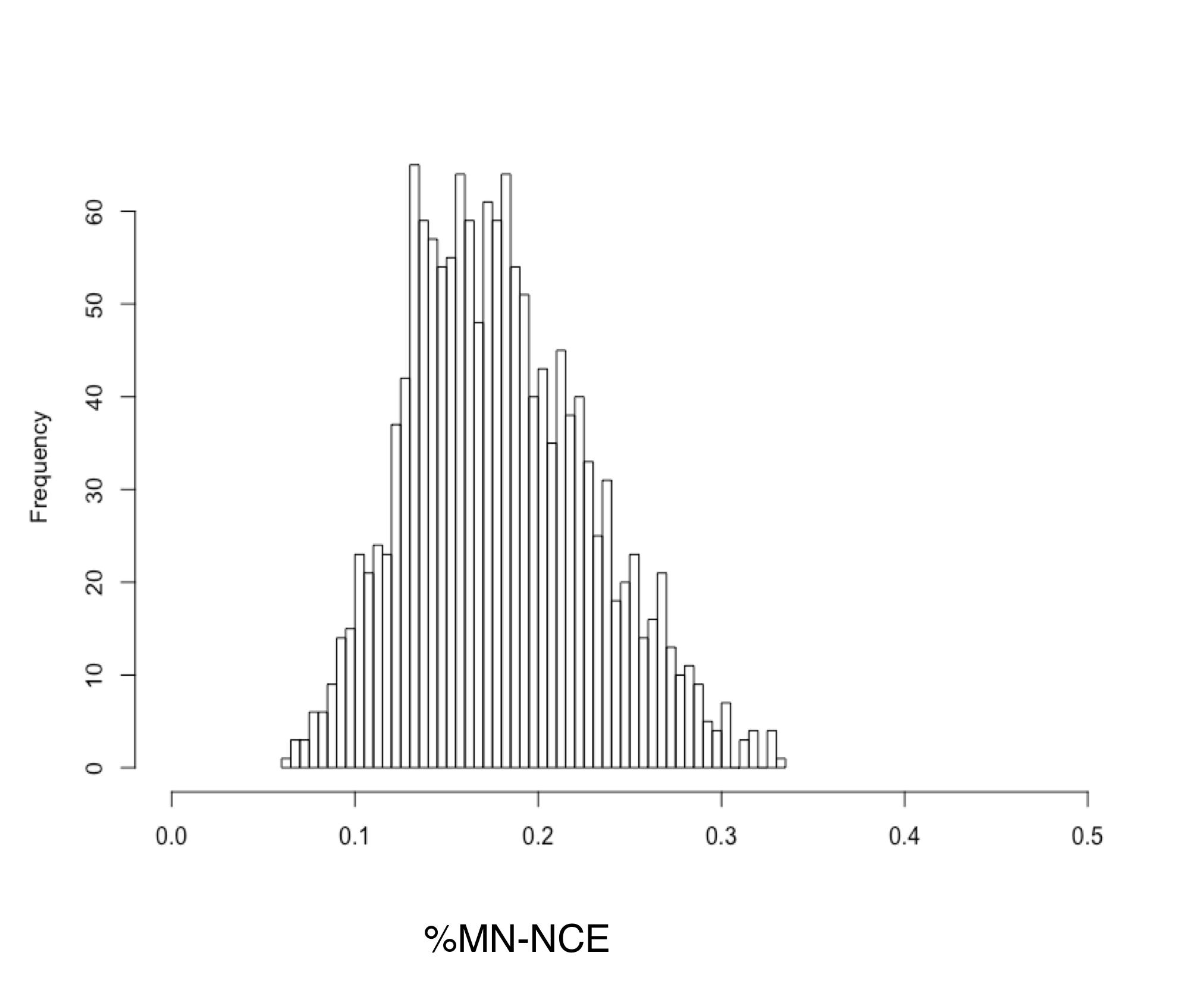

Supplement: Supplemental Material [file supp_g3.116.030767_FigureS1.tif]

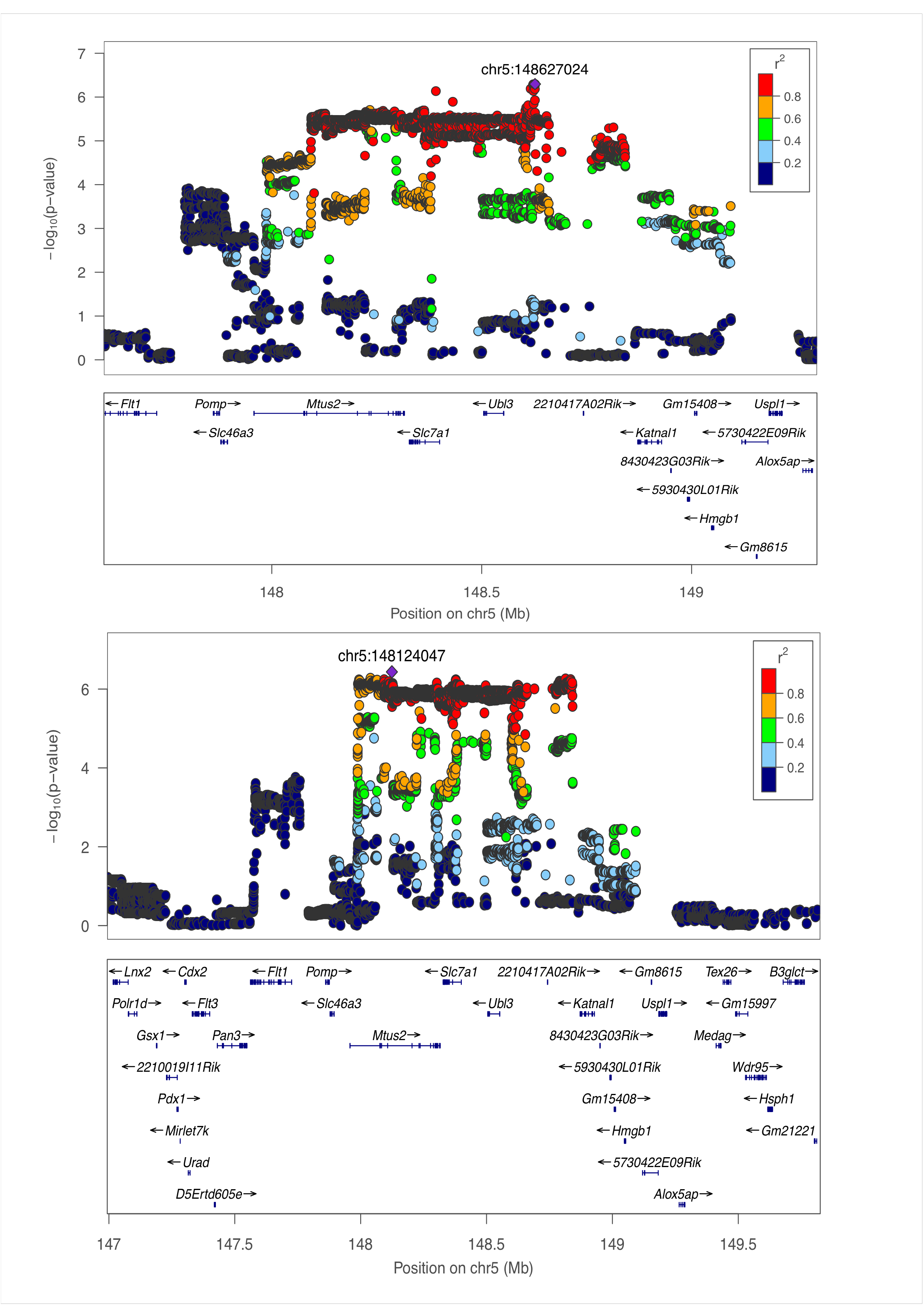

Supplement: Supplemental Material [file supp_g3.116.030767_FigureS2.tif]
